# Deferoxamine Inhibits Acute Lymphoblastic Leukemia Progression through Repression of ROS/HIF-1*α*, Wnt/*β*-Catenin, and p38MAPK/ERK Pathways

**DOI:** 10.1155/2022/8281267

**Published:** 2022-02-21

**Authors:** Hongliang You, Dao Wang, Linlin Wei, Jiao Chen, Huanhuan Li, Yufeng Liu

**Affiliations:** Department of Hematology and Oncology, Children's Hospital, The First Affiliated Hospital of Zhengzhou University, Zhengzhou, China

## Abstract

Acute lymphoblastic leukemia (ALL) is the most common type of childhood cancer, with a feature of easy to induce multidrug resistance and relapse. Abundant studies have proved that iron overload strengthens the growth and metastasis of tumor cells. Herein, we found that deferoxamine (DFO) effectively decreased the concentration of intracellular iron in ALL cells. DFO inhibited proliferation, induced apoptosis, and obstructed cell cycle of ALL cells, whereas DFO and dextriferron (Dex) used in combination significantly decreased the sensitivity of ALL cells to DFO. Reactive oxygen species (ROS) level was reduced in ALL cells treated with DFO, and the combination of DFO and Dex reversed the effects of DFO. *In vivo*, DFO inhibited mouse tumor growth. Besides, cyclinD1, *β*-catenin, c-Myc, hypoxia inducible factor 1 (HIF-1), p-p38MAPK, and p-ERK1/2 protein levels were significantly downregulated, and the levels of prolyl hydroxylase-2 (PHD-2) were upregulated after treated with DFO, whereas Dex treatment reversed those *in vivo* and *in vitro*. In conclusion, DFO inhibited the proliferation and ALL xenograft tumor growth, obstructed the cell cycle, and induced apoptosis of ALL cells, probably via inactivating the ROS/HIF-1*α*, Wnt/*β*-catenin, and p38MAPK/ERK signaling.

## 1. Introduction

Acute lymphocytic leukemia (ALL) is a rare hematological malignancy, contributing to about 20% of new acute leukemias [1]. In the United States, in spite of aggressive first-line treatment, the 5-year overall survival rates of the ALL patients of 40–59- and 60–69-years-old are 24% and 18%, respectively [2]. Recent advances of chemotherapy result in a good chance (approximately 85%) for a cure in children who are newly diagnosed with ALL [3]. However, the remaining 15% of ALL children cannot survive due to multidrug resistance and relapse [4]. Therefore, there is an urgent need for new treatment methods to improve the survival rate of ALL patients who have failed treatment or relapsed after the initial response.

Studies have shown that ALL is related to excess iron in the body [5]. Iron is an important nutrient and regulator for physiological processes in cells. Iron can catalyze metabolic activities, which is essential to cell survival and proliferation. According to reports, many forms of cancer are associated with massive iron overload, possibly due to the need for iron as a cofactor to maintain the tumor growth and proliferation [6, 7]. Iron deficiency reduced the aggressiveness of a variety of tumor cells, which has important clinical value in the treatment of leukemia [8, 9]. Iron chelator triapine has been reported to be able to inhibit the ribonucleotide reductase activity and tumor growth both *in vivo* and *in vitro* and has been involved in the phase I and phase II clinical trials [10]. Besides, some other iron-chelating agents also have potential antitumor activity, such as deferasirox [11], DpC [12], Dp44 mT [13], gold tricarboxylic acid (ATA), and deferoxamine (DFO) [14]. According to the reports of Benadiba et al. [15], DFO has significantly affected the survival of T lymphoblastic leukemia/lymphoma cells to induce apoptosis and demonstrated synergistic action with three ALL-specific drugs: L-asparaginase, doxorubicin, and dexamethasone.

Therefore, as a new type of antitumor drug, iron chelator has attracted wide interest from scholars, but its specific roles and potential mechanism by either disrupting intracellular iron homeostasis or depleting intracellular iron in acute lymphoblastic leukemia have not been fully elucidated. Here, we assessed the tumor-suppressive effect and underlying mechanism of DFO on ALL via targeting iron metabolism. Our findings cast light on the development of more effective treatments for acute lymphoblastic leukemia.

## 2. Materials and Methods

### 2.1. Cell Lines and Cell Culture

The acute lymphoblastic leukemia cell lines Jurkat and NALM-6 were afforded from the American Type Culture Collection (ATCC, USA) and incubated using the RPMI-1640 (Sigma-Aldrich, Grand Island, NY, USA)) comprised of 10% heat-inactivated fetal bovine serum (FBS; GIBCO, MA, USA) and 50 units/ml penicillin and 500 mg/ml streptomycin (GIBCO, USA). All cells were constantly maintained in a humid atmosphere containing 5% CO_2_ at 37 °C.

### 2.2. CCK-8 Assays

Viability of ALL cells was assessed using the CCK-8 assay. Briefly, NALM-6 and Jurkat cells (2 × 10^4^ cells/well) were cultured in 96-well plates with 0, 5, 10, 50, 100, and 150 *μ*mol/L deferoxamine (DFO) (Amadis Chemical Company Limited, Shanghai, China) and incubated for 48 h at 37°C and 5% CO_2_. Then, 10 *μ*L of CCK-8 (SevenBio, CA, USA) was aseptically added and incubated for another 4 h. Subsequently, the optical density (OD) values were calculated at the absorbance of 450 nm via a Varioskan™ LUX multifunction microplate reader (Thermo Fisher Scientific, Waltham, MA, USA).

For detecting antiproliferative effects of DFO, ALL cells (3 × 10^3^ cells/well) were cultured for the indicated times (0, 1, 2, and 3 days). After incubation, the CCK-8 assay was performed with standard procedures.

### 2.3. Detection of Intracellular Labile Iron Pool (LIP) Level

The calcein-AM assay was used to detect the concentration of intracellular iron of ALL cells. Briefly, the NALM-6 and Jurkat cells (2 × 10^5^ cells/well) were cultured with or without DFO (100 *μ*mol/L) and dextriferron (Dex, 20 *μ*g/mL) (Sigma-Aldrich, St. Louis, MO, USA). After 24 h, cells were labeled by the final concentration of 0.125 *μ*mol/L calcein-AM (Aladdin Bio-Chem Technology, Shanghai, China) for another 10 min. These cells were then centrifuged and washed twice to remove unbound fluorescent indicators and resuspended in PBS. Trypan blue with a concentration of 0.25 *μ*g/mL was added to quench the extracellular fluorescence, and the fluorescence intensity of cells was detected by using a fluorescence spectrophotometer. Thereafter, iron-chelating agent 2,2-bipyridyl (BIP) with a final concentration of 100 *μ*mol/L was added and treated for 30 min. Then, fluorescence intensity of cells was detected by using a fluorescence spectrophotometer and the excitation (Ex) wavelength was set at 495 nm and the emission wavelength (Em) was set at 530 nm. The difference of fluorescence intensity between the two detection results was calculated, which represented the content of intracellular calcein binding iron, namely the intracellular LIP level.

### 2.4. Apoptosis Analysis

The NALM-6 and Jurkat cells (1 × 10^6^ cells/well) were incubated with DFO alone or in combination with Dex for 48 h. After the cells were collected and washed and resuspended with cold PBS, an annexin V-PE/7-ADD apoptosis detection kit (BD Pharmingen™, CA, USA) was employed to detect the apoptosis in ALL cells. Eventually, total cell apoptosis was assessed using a Becton–Dickinson FACScan (BD Corporation, USA). Data were analyzed using the E Cell Quest software version 2.0 (BD Corporation, USA).

### 2.5. Cell Cycle Analysis

The NALM-6 and Jurkat cells (1 × 10^6^ cells/well) were incubated with DFO alone or in combination with Dex for 48 h. For the cell cycle assay, the harvested cells were fixed with 75% ethanol at −20 °C overnight. The cells were stained with 400 *μ*L of PI (20 *μ*g/ml) and 100 *μ*L of RNase A (100 *μ*g/mL) for 30 min. Cell cycle was detected using flow cytometry and analyzed via the ModFit analysis software (Verity Software House, ME, USA).

### 2.6. Reactive Oxygen Species (ROS) Analysis

The NALM-6 and Jurkat cells (2 × 10^6^ cells/mL) were collected to measure the levels of ROS. DCFH-DA (10 *μ*M) was added into cell suspension, and the cells were incubated for 30 min at 37 °C in dark. Then, the cells were washed twice with PBS, and the levels of ROS were evaluated on a Becton–Dickinson FACScan flow cytometer at 488 nm.

### 2.7. In Vivo Experiment

Thirty BABL/c nude female mice (5 weeks, 18–22 g) were obtained from the Animal Center of the Chinese Academy of Sciences and maintained in a temperature- and light-controlled environment with free access to water and food. NALM-6 cells (4 × 10^6^/200 *μ*L/mouse) in a serum-free medium were hypodermically injected into the flank of the nude mouse for establishing the acute lymphocytic leukemia models. Mice were distributed randomly into 3 treatment groups and were intraperitoneally given saline, DFO (50 mg/kg/day), and (DFO 50 mg/kg + Dex 5 mg/kg/d) once a day for 34 days. The tumor weight and volumes were measured and calculated every week. After 35 days, the treated mice were sacrificed and the tumors were dissected and assessed. All mice experiments were performed in compliance with the animal protection legislation and approved by the Animal Experimentation Ethics Committees of Zhengzhou University (approval number: 2018-KY-51).

### 2.8. Western Blot Analysis

Collected samples were lysed with ice-cold RIPA lysis buffer (Sigma) supplemented with protease inhibitors (Thermo Fisher). The concentration of extracted proteins was quantified by the Braford method (BioRad, Amersham, Sweden). The equal protein lysates were denatured in SDS sample buﬀer and separated by 10% SDS-PAGE followed by electrotransfer onto the nitrocellulose membrane. Membranes were blocked with 5% skimmed milk powder for 30 min and incubated with primary antibodies (against Ki67, Bid, Bcl-xl, GAPDH, *β*-catenin, cyclinD1, c-Myc, HIF-1*α*, PHD-2, p38MAPK, p-p38MAPK, p-ERK1/2, and ERK1/2) overnight at 4°C and subsequently hatched with the matching secondary antibodies at room temperature for 2 h. Protein signals were visualized by using ECL reagent and exposed to the ChemiDoc XRS system (Thermo, Bethesda, MD, USA).

### 2.9. Statistical Analysis

All data were collected from three separate experiments, and values were expressed as mean ± SD. Analyses were conducted using the SPSS 17.0 software. Differences between two groups were evaluated using Student's *t*-test, and multiple comparisons were conducted by one-way analysis of variance (ANOVA). Differences were statistically significant when the *P* value < 0.05.

## 3. Results

### 3.1. DFO Inhibits Cell Viability of Jurkat and NALM-6 Cells by Reducing Iron Concentration

**Figure 1:**
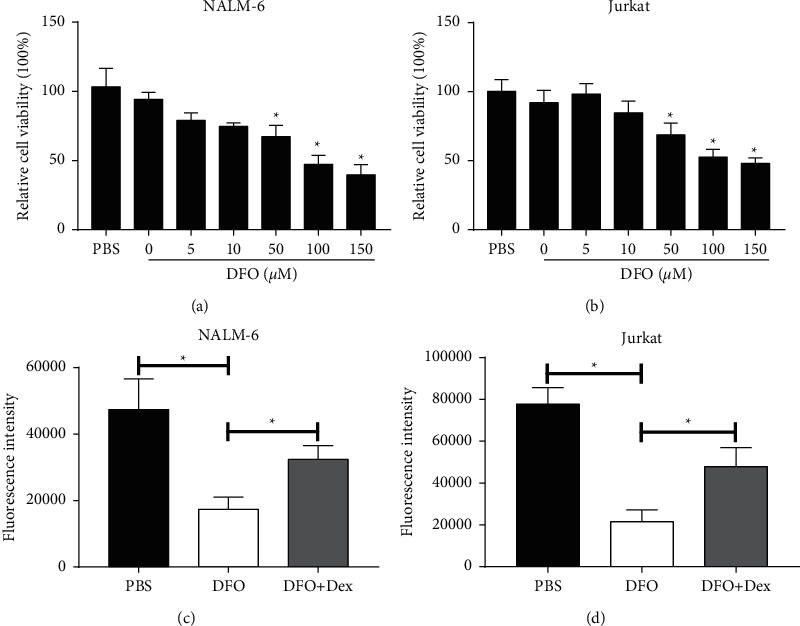
DFO inhibits the cell viability of Jurkat and NALM-6 cells. (A, B) Cells were treated with DFO (0, 5, 10, 50, 100, and 150 *μ*mol/L) for 48 h. Proliferative viability was measured by CCK-8 assays. (C, D) Intracellular iron concentration was determined by the calcein-AM assay after DFO treatment on ALL cell lines for 24 h. Data are presented as mean ± SD, *n* = 3. ^*∗*^*P* < 0.05.

As demonstrated in Figures 1(a) and 1(b), DFO significantly inhibited the viability of Jurkat and NALM-6 cells in a concentration-dependent manner. Then, we chose DFO (100 *μ*mol/L) for subsequent functional analyses. To examine the abundance of cellular labile iron pool in the ALL cell lines Jurkat and NALM-6 cells, the calcein-AM assay suggested that Dex effectively increased the concentration of intracellular iron, whereas DFO effectively decreased the concentration of intracellular iron in Figures 1(c) and 1(d).

### 3.2. DFO Inhibits Cell Proliferation of NALM-6 and Jurkat Cells

**Figure 2:**
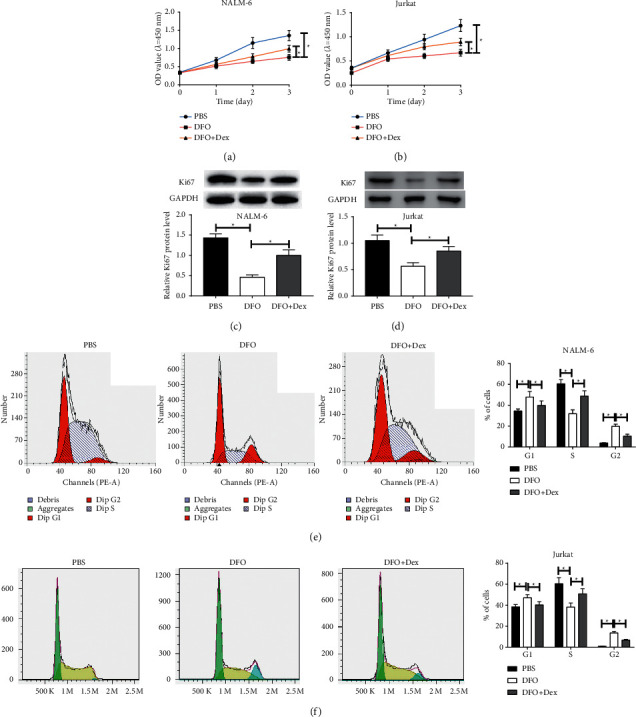
The effect of DFO on cell proliferative activity and cell cycle of Jurkat and NALM-6 cells. (A, B) Proliferation of cells treated with DFO (100 µmol/L), or DFO (100 µmol/L) and Dex (20 µg/mL) in combination was measured by CCK-8 assays after culturing for indicated time. (C, D) ALL cells were treated with DFO (100 µmol/L) or DFO (100 µmol/L) and Dex (20 µg/mL) in combination for 48 h. Western blot analyses for expression of Ki67 in ALL cells. (E, F) At 48 h after indicated treatments (DFO (100 µmol/L), or DFO (100 µmol/L) and Dex (20 µg/mL)), cell cycle distribution of Jurkat and NALM-6 cells was determined by the flow cytometry assay. Data are presented as mean ± SD, *n* = 3. ^*∗*^*P* < 0.05.

To evaluate the potential effect of DFO on the proliferation of ALL cells, the CCK-8 assay was also implemented. The proliferation activity of NALM-6 and Jurkat cells subjected to DFO (100 *μ*mol/L) was suppressed in comparison with the control, while the DFO (100 *μ*mol/L) and Dex (20 *μ*g/mL) cotreated group inversed the inhibition effect in comparison to the DFO group (Figures 2(a) and 2(b)). Western blot analyses showed that Ki67 was reduced in the DFO-treated group compared with the control group but increased in the combination group compare to the DFO single-treatment group (Figures 2(c) and 2(d)), which was in concordance with the results of the CCK-8 assay. Moreover, in order to investigate the effects of DFO on the cell cycle of NALM-6 and Jurkat cells, the flow cytometry assay was performed. DFO reduced the number of ALL cells at S stage, while the DFO and Dex cotreated group attenuated the effect (Figures 2(e) and 2(f)). Altogether, DFO suppresses the proliferative ability in ALL cells, whereas Dex attenuated the inhibition.

### 3.3. DFO Promotes Apoptosis in ALL Cells

**Figure 3:**
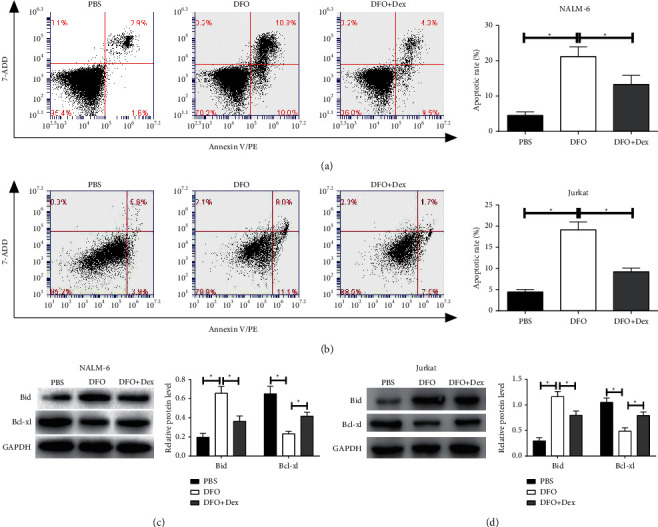
The effect of DFO on cell apoptosis in Jurkat and NALM-6 cells. (A, B) The cells were treated with DFO, or DFO and Dex in combination at indicated concentrations at 48 h, and the apoptosis rate was assessed by the flow cytometry assay. (C, D) The levels of Bid and Bcl-xl were analyzed by western blot. Data are presented as mean ± SD, *n* = 3. ^*∗*^*P* < 0.05.

Abnormal growth of tumor cells may be closely related to apoptosis [16]. The DFO significantly promoted the apoptosis rates in NALM-6 and Jurkat cells, whereas Dex inhibited the increased apoptosis induced by the DFO treatment compared with the DFO single treatment in ALL cells (Figures 3(a) and 3(b)). The expression of Bid and Bcl-xl was detected by western blot assays to explore the underlying mechanism of DFO on regulating the apoptosis of ALL cells. As shown in Figures 3(c) and 3(d), DFO elevated the levels of Bid and decreased the levels of Bcl-xl in comparison with the control treatment, whereas the DFO and Dex combination treatment reversed the effect of DFO treatment in ALL cells (Figures 3(c) and 3(d)).

### 3.4. DFO Inhibits ROS Production in ALL Cell Lines

**Figure 4:**
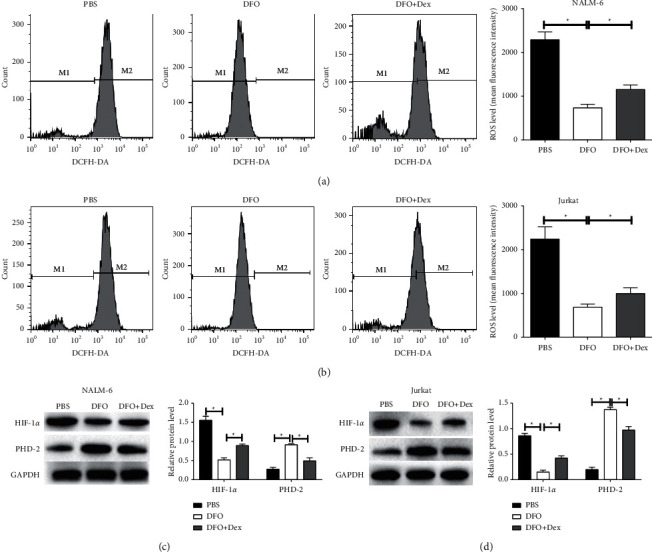
The effects of DFO on ROS production in Jurkat and NALM-6 cells. (A, B) The ROS levels in cells treated with DFO, or DFO and Dex in combination were detected by flow cytometry assays. (C, D) Western blot assays for detecting the HIF-1*α* expression and PHD-2 expression in cells. Data are presented as mean ± SD, *n* = 3. ^*∗*^*P* < 0.05.

To further explore the effects of DFO on ROS production in ALL cells, flow cytometry assays were performed. As shown in Figures 4(a) and 4(b), the results revealed that DFO treatment significantly reduced the levels of ROS and Dex reversed its effects. Furthermore, western blot results showed that HIF-1*α* expression was obviously inhibited in ALL cells treated with DFO, and PHD-2 expression was significantly increased, whereas Dex reversed those effects of DFO (Figures 4(c) and 4(d)). These results suggested that DFO inhibited ROS production by repressing the HIF-1*α*/PHD-2 pathway in ALL cells.

### 3.5. DFO Inhibits the Wnt/*β*-Catenin and ERK Cascades in ALL Cell Lines

**Figure 5:**
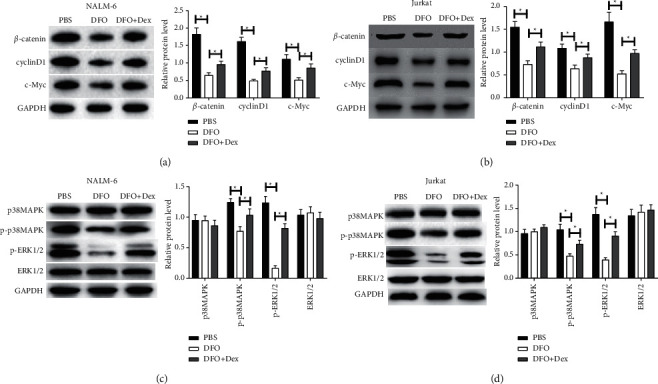
Effect of DFO on Wnt/*β*-catenin and p38MAPK/ERK pathways in NALM-6 and Jurkat cells. (A, B) The protein expression of *β*-catenin, cyclinD1, and c-Myc was detected by western blot. (C, D) The protein expression of p-p38MAPK, p38MAPK, ERK1/2, and p-ERK1/2 was measured by western blot. Data are presented as mean ± SD, *n* = 3. ^*∗*^*P* < 0.05.

As shown in Figures 5(a) and 5(b), the protein expression levels of c-Myc, cyclinD1, and *β*-catenin were lowered after DFO treatment, whereas Dex inverted this affection. In addition, as shown in Figures 5(c) and 5(d), DFO significantly downregulated p-ERK1/2 and p-p38MAPK compared with the control. Moreover, p-p38MAPK and p-ERK1/2 were upregulated in the DFO and Dex cotreated group compare to the DFO-treated group. All results indicated that that DFO exerted antiproliferation, obstructed cell cycle, and induced apoptosis in ALL cells probably involved in the Wnt/*β*-catenin and p38MAPK/ERK cascades.

### 3.6. DFO Inhibits ALL Xenograft Growth

**Figure 6:**
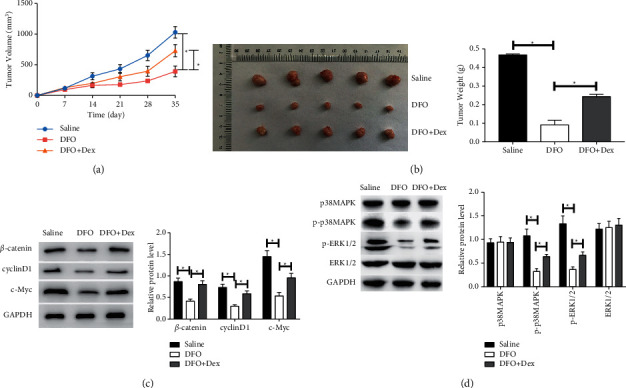
Effect of DFO on the growth of NALM-6 xenograft. (A) The effect of DFO on the tumor volumes in xenograft mouse. (B) Tumor weight was calculated. (C, D) The protein expression of *β*-catenin, cyclinD1, c-Myc, p-p38MAPK, p38MAPK, ERK1/2, and p-ERK1/2 in NALM-6 xenograft tissues by western blot. Data are presented as mean ± SD, *n* = 5. ^*∗*^*P* < 0.05.

To further validate the impact and the underlying mechanism of DFO *in vivo*, NALM-6 xenograft mice were acquired by hypodermical injection of NALM-6 cells. The xenograft tumor volume and weight in the DFO group were decreased compared to the saline group, while the DFO and Dex combination group significantly reduced the change induced by DFO (Figures 6(a) and 6(b)). Moreover, the western blot assay showed that DFO treatment resulted in a decrease in the protein levels of *β*-catenin, cyclinD1, c-Myc, p-p38MAPK, and p-ERK1/2 compared to the saline treatment in the tumor tissues, whereas DFO and Dex treatment in combination alleviated the abovementioned observations (Figures 6(c) and 6(d)). Taken together, these results further confirmed that DFO significantly exerted inhibition of ALL via reducing intracellular iron levels *in vivo* and *in vitro*.

## 4. Discussion

In the United States alone, about 3000 children and teenagers are diagnosed with acute lymphocytic leukemia (ALL) each year [17]. Iron is vital for the physiological function of cells [18]. The abnormal iron metabolism and iron homeostasis disorders have been considered as a vital factor of the pathophysiology of various diseases such as cancer, neurodegenerative disease, cardiovascular complications, and infections [19]. Studies have shown that excessive iron can induce tumor and promote tumor growth, whereas iron deprivation can suppress tumor cell growth and promote apoptosis, and the degree of influence varies with the dose and the effect time of iron deprivation agent [20]. The study by Becton et al. [21] has shown that DFO has antitumor activity against malignant cells of patients with leukemia and acute lymphocytic leukemia, acute myeloid leukemia, and neuroblastoma. DFO is widely used for the treatment of iron overload diseases, and its antitumor effect has attracted more and more attention.

Iron-chelating agents exhibit potential as a new therapy for cancer. In recent years, more and more research studies have been conducted on the antileukemia effect of iron chelators. For example, Chang et al. have reported that deferasirox has strong antileukemia activity [22]. A large number of *in vitro, in vivo*, and clinical studies reveal that iron-depleting drugs can suppress the aggressiveness of tumor cells and tumor xenografts via chelating intracellular molecules and regulating the levels of iron-regulatory protein and has obvious antiproliferative effects in cancer, which has provided a new direction for anticancer therapies [23, 24]. The study by Lee has verified that iron-chelating agents either deferasirox (DFX) or deferoxamine (DFO) could reduce the survival ability of leukemic cells in a dose-dependent manner [25]. Interestingly, our results demonstrated that DFO reduced the proliferation of NALM-6 and Jurkat cells and growth of tumor xenografts, whereas Dex attenuated the inhibition, indicating that iron-chelating agent DFO could antagonize iron load and suppress the proliferation and growth of ALL cells and tumor xenografts.

Studies have shown that iron chelation can be used to inhibit the abnormal proliferation of tumor cells for the reason that depletion of iron exhibits antitumor effects [26]. Yang et al. have shown that iron chelator inhibits the proliferation of leukemia cells and induces apoptosis by regulating the expression of apoptosis-related genes [27]. The study of Tomoyasu et al. has exhibited that iron-chelating agent can induce HL-60 and K562 apoptosis and the mechanism is probably by arresting cells growth at the S-phase [28]. Here, we observed that DFO promoted apoptosis via regulating the expression of proapoptotic Bid and apoptotic suppressor Bcl-xL and obstructed the cell cycle of ALL cell lines, while these effects were further abrogated when the iron levels increased. Iron is reported to be able to result in the abnormal production of ROS in ALL cells [29]. In addition, a reported research has shown that iron promotes the carcinogenesis by inducing the production of ROS through activating JNK and MAPK pathways [30]. In this study, DFO reduced the ROS levels in ALL cells, and the effects were reversed by Dex, which suggests that iron reduction by DFO confers the inhibitory effects on ROS production. HIF-1*α*, an important factor, is involved in cell oxidative stress and inflammation [31]. We found that the levels of HIF-1*α* were reduced in ALL cells treated with DFO, and prolyl hydroxylase domain enzyme-2 (PHD-2) expression was increased, whose effects were reversed by Dex treatment. Our results were consistent with the reported research by Janssens et al. [32]. They have confirmed that higher levels of HIF-1*α* are expressed in MDS-MSC of the iron-overload (IO) group than those in the non-iron overload (NIO) group and high levels of ROS induce the HIF-1*α* level to increase through inhibiting the PHD-2 in MDS-MSC. These results suggested that iron depletion by DFO inhibited ROS production by repressing the HIF-1*α*/PHD-2 pathway in ALL cells. However, the previous study has suggested a reverse effect which is that DFO induces the increase of HIF-1*α* and DFO-induced leukemic cell apoptosis by mitochondrial pathway-dependent and HIF-1*α*-independent mechanisms [33]. These results suggest that DFO-induced apoptosis and DFO-induced inhibition of proliferation and ROS may involve in different initiating signal pathways in different types of cells that remain to be explored.

The Wnt/*β*-catenin signaling regulates the transcription of various oncogenes, including cyclin c-Myc, cyclinD1, and Bcl-w [34]. Abnormal activation of this signaling pathway has been shown in various cancers, such as colorectal cancer, lung cancer, breast cancer, ovary cancer, prostate cancer, synovial sarcoma, and Schwann cell tumors [35]. The widespread loss of control of the Wnt signaling in various malignancies makes it an attractive therapeutic target [36]. Research on targeting Wnt/*β*-catenin signal transduction in cancers related to Wnt activation has opened up a new avenue for the development of effective drugs to inactivate the Wnt/*β*-catenin signaling [37].

Abnormal activation of the Wnt/*β*-catenin signaling correlates with the prognosis of patients suffering acute myeloid leukemia (AML) [38]. In addition, the Wnt/*β*-catenin signaling is necessary for the development of highly proliferative leukemia stem cells (LSCs), which is believed to be able to maintain the survival of leukemia [39]. Furthermore, iron load in colorectal cancer cells can promote the Wnt signaling and cell proliferation [40], but iron chelators, including deferoxamine, deferoxamine, and ciclopirox, can inactivate the Wnt signaling, and AML patients taking cyclohexanolamine show a significant decrease in the expression of the Wnt target gene AXIN2. A reported study [41] has shown that depletion of iron weakens the Wnt signaling pathway. Song et al. [42] have reported that acyl compounds inhibit the stability of *β*-catenin, prevent the transcription induced by Wnt signaling, and further block the growth of cancer cells through binding to iron. Collectively, the protein expression levels of c-Myc, cyclinD1, and *β*-catenin were lower in DFO-treated cells and tumor xenografts. However, the Dex and DFO cotreated group alleviated the abovementioned observations, suggesting that DFO inhibits the ALL tumor probably through inhibiting the Wnt/*β*-catenin signaling, which probably serves as a major mechanism in iron chelator-mediated cell death of ALL cells by activating downstream apoptotic cascade.

Accumulating evidence suggests that iron chelators influence the MAPK cascades, via dephosphorylate MAPKs, such as MAPK/P38 kinase, ERK, and c-Jun N-terminal kinase (JNK) [43], which are at the center of signaling crosstalk of lymphoblastic activation [44]. Lee et al. [45] have demonstrated that DFO can inhibit the growth and promote apoptosis of IHOK and HN4 cells in a time-dependent and dose-dependent manner via activating p38 MAP kinase and ERK, all of which act as a downstream apoptotic cascade that conducts the iron chelator-mediated cell death mechanism. Studies by Kim et al. [46] have reported that p38 MAP kinase and extracellular ERK are activated by chelator DFO at an early stage of incubation. However, the data of Haigl et al. [47] unveil that DFO and hypoxia treatment increases Spry4 expression, accompanied with inhibiting the activation of ERK signaling. Similarly, our current study revealed that DFO markedly reduced the phosphorylation of p38MAPK and ERK, indicating that p38MAPK/ERK arrested the cell cycle and induced apoptosis by iron depletion.

## 5. Conclusion

In summary, DFO reduced the proliferation and tumor growth, obstructed the cell cycle and ROS production, and induced apoptosis of ALL cell lines by inactivating the HIF-1*α*/PHD-2, Wnt/*β*-catenin, and p38MAPK/ERK pathways by iron depletion. Current results seem likely to generate an interesting adjuvant therapy for the acute lymphocytic leukemia and identify potential targets for anticancer therapy.

## Data Availability

All the data generated or analyzed during this study are included within this article.
